# Genome-wide association study and gene network analyses reveal potential candidate genes for high night temperature tolerance in rice

**DOI:** 10.1038/s41598-021-85921-z

**Published:** 2021-03-24

**Authors:** Raju Bheemanahalli, Montana Knight, Cherryl Quinones, Colleen J. Doherty, S. V. Krishna Jagadish

**Affiliations:** 1 International Rice Research Institute, DAPO Box 7777, Metro Manila, Philippines; 2 Department of Agronomy, 2004 Throckmorton Plant Sciences Center, Kansas State University, 1712 Claflin Road, Manhattan, KS 66506-5501 USA; 3 Department of Molecular and Structural Biochemistry, North Carolina State University, Raleigh, NC 27695 USA; 4 Present Address: Arkansas Biosciences Institute, Arkansas State University, P. O. Box 419, Jonesboro, AR 72467 USA; 5 Present Address: Department of Plant and Soil Sciences, Mississippi State University, 117 Dorman Hall, Box 9555, Mississippi State, MS 39762 USA

**Keywords:** Agricultural genetics, Plant stress responses

## Abstract

High night temperatures (HNT) are shown to significantly reduce rice (*Oryza sativa* L.) yield and quality. A better understanding of the genetic architecture of HNT tolerance will help rice breeders to develop varieties adapted to future warmer climates. In this study, a diverse *indica* rice panel displayed a wide range of phenotypic variability in yield and quality traits under control night (24 °C) and higher night (29 °C) temperatures. Genome-wide association analysis revealed 38 genetic loci associated across treatments (18 for control and 20 for HNT). Nineteen loci were detected with the relative changes in the traits between control and HNT. Positive phenotypic correlations and co-located genetic loci with previously cloned grain size genes revealed common genetic regulation between control and HNT, particularly grain size. Network-based predictive models prioritized 20 causal genes at the genetic loci based on known gene/s expression under HNT in rice. Our study provides important insights for future candidate gene validation and molecular marker development to enhance HNT tolerance in rice. Integrated physiological, genomic, and gene network-informed approaches indicate that the candidate genes for stay-green trait may be relevant to minimizing HNT-induced yield and quality losses during grain filling in rice by optimizing source-sink relationships.

## Introduction

Rice is a major source of calories for more than 50% of the world’s population and plays a key role in sustaining food security in Asia^1^, Africa, and the Americas^2^. Current global climate models predict a mean temperature increase by 1.0–3.7 °C by 2100^3,4^, which would potentially increase the occurrence of heat stress events in rice-growing tropical and subtropical countries. Disaggregating the predicted mean temperature increase has allowed unraveling a rapid rise in night temperature compared to the maximum day temperature at the farm^5,6^, region^7,8^, and global^9^ scale. A rapid rise in night temperature is fast becoming a significant challenge for sustaining crop yields, including rice^5,10–13^, wheat (*Triticum aestivum*)^14,15^ and barley (*Hordeum vulgare*)^16,17^. Previous studies have shown that an average season-long increase in minimum night temperature by 1 °C reduced tropical irrigated rice (*Oryza sativa* L.) grain yield by 10%^5^. Additionally, field-based studies document the susceptibility of rice inbred cultivars or commercial hybrids exposed to HNT (28 or 29 °C), leading to an average grain yield reduction of ~ 15%^13,18^ to 90% when exposed to severe HNT (32 °C) under controlled environments^19^. An increase in night temperatures have resulted in a negative impact on grain yield and quality in the major rice-growing regions of South and Southeast Asia^5,7^ and the United States^20^. Therefore, breeding programs need to focus on enhancing high night temperature (HNT) stress tolerance in rice to minimize yield and grain quality losses.

Unlike the pre-flowering phase, the post-flowering phase is most affected by HNT, wherein the rate of senescence is advanced leading to lower grain-filling duration, resulting in lower grain yield and quality^13,14^. In response to high day temperature (HDT), rice can benefit from early morning flowering mechanism to escape or effectively employ transpiration cooling to avoid heat stress during the day^21,22^. However, rice is not equipped with similar physiological processes that can minimize the negative impact of HNT^22–24^. Interestingly, the physiological routes through which HDT and HNT stress induce yield losses are shown to differ, although both the stresses finally result in significant yield and quality losses^22^. Experiments under controlled environments^25–29^ and field conditions^30,31^ have convincingly demonstrated that HDT stress negatively impacts reproductive processs in rice, leading to spikelet sterility. However, induction of HNT from panicle initiation until physiological maturity under field conditions did not induce a significant reduction in spikelet sterility and had no loss in seed number^11^. The same response was captured even with different levels of nitrogen application^11,32^ and across a range of inbreds and hybrids^13,18,33^, providing strong support for the differential route through which HNT induced yield and quality losses in rice. These results make a strong case that progress achieved through genetic mapping for HDT stress in rice may not be directly translated to induce HNT tolerance in rice^22^.

Rice exposed to HNT stress during the grain-filling stage significantly reduces whole plant and panicle carbohydrate content due to increased night respiration (carbon loss) resulting in lower biomass, lower grain yield (due to lower 100-seed weight), and enhanced chalkiness^11,13,15,34^. Grain yield and quality, specifically chalkiness, are key traits that drive market value and are considered significant aspects for assessing HNT impact in rice^10,13^. Despite the increasing knowledge of the metabolomic^35^ and transcriptomic^36,37^ responses of rice to HNT, our understanding of the genetic basis of rice response to HNT tolerance is mostly unexplored. Hence, to the best of our knowledge, this is the first study to identify genetic loci and putative candidate genes controlling HNT response in rice that will help in developing HNT tolerant varieties and hybrids for current and future warmer climates.

To fill the above knowledge gaps, an *indica* panel was phenotyped for grain yield, 100-seed weight, harvest index, grain size and chalkiness, under HNT using controlled environment walk-in chambers and field-based heat tents. The diversity panel consisting of improved and traditional genotypes with sub/tropical adaptation was phenotyped to (1) identify genomic regions associated with phenotypic traits in control and HNT in controlled environments and field conditions; (2) compare the extent of co-localization of the genetic loci across treatments under controlled environment and field conditions, and (3) identify putative candidate genes associated with enhanced HNT tolerance in rice using gene network analysis.

## Materials and methods

### Plant materials

The *indica* rice diversity panel (PRAY—Phenomics of Rice Adaptation and Yield) involving cultivars from tropical and subtropical rice-growing regions were assembled as a part of the Global Rice Phenotyping Network (GRPN, http://ricephenonetwork.irri.org). A sub-set of two-hundred and nine genotypes was chosen based on the uniformity in flowering phenology, using data generated under the International Rice Research Institute (IRRI) farm conditions^38^. Two independent experiments were conducted during 2015 (Experiment 1—controlled environment facility) and 2016 (Experiment 2—field-based heat tents) at IRRI, Los Baños (14° 11′ N, 121° 15′ E, 21 masl), Philippines. In Exp. 1, the 209 genotypes with 3 replicates and two treatments resulted in 1254 pots. In Exp. 2, the same 209 genotypes with each grown in a row (each row having 15 plants) and two treatments resulted in about 6270 plants (Supplementary Fig. S1). Plants in both experiments were grown under fully flooded conditions to study the effect of HNT stress starting from panicle initiation until physiological maturity. Unlike short episodes of high day heat stress, HNT is acknowledged to persist for longer growth periods encompassing different developmental stages, leading to yield and quality losses^11,13^. Previous studies using multiple genotypes with HNT stress imposed in the same field-based tents concluded that the impact of HNT was significant when coincided with panicle initiation and lasting until physiological maturity, but not during the early vegetative stage^33^.

## Experiment 1 (walk-in chamber facility)

### Crop management

**Figure 1:**
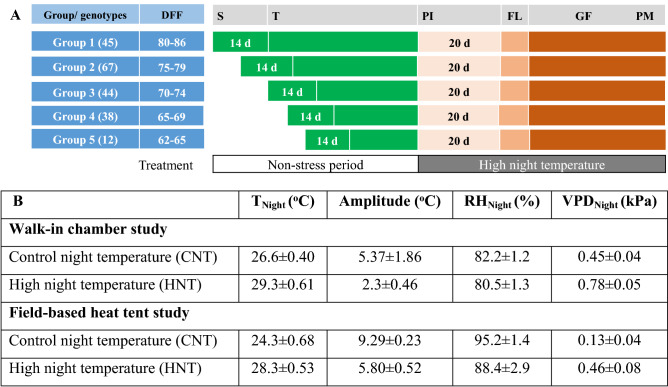
Synchronization of the reproductive stage by stagger sowing (**A**) for phenotyping *indica* panel under HNT stress (**B**) in walk-in chambers (Exp. 1) and field-based heat tents (Exp. 2). *DFF* days to 50% flowering, *S* seeding, *T* transplanting, *PI* panicle initiation, *FL* flowering, *GF* grain filling, *PM* physiological maturity. Amplitude is calculated as a difference between average day temperature and average night temperature for each treatment separately. *VPD* vapor pressure deficit, *RH* relative humidity and *SD* standard deviation. Schematic representation of materials and methods followed to impose HNT stress is visualized in Supplementary Fig. S1.

Seed dormancy of rice genotypes was broken by exposing them to 50 °C for three days and were then hand sown in seeding trays. Fourteen-day-old seedlings were transplanted into plastic pots (25 cm in height; 26 and 20 cm diameter at the top and bottom, respectively), filled with 6 kg of clay loam farm soil and 2.0 g ammonium sulfate [(NH_4_)_2_SO_4_], 1.0 g single superphosphate (SSP) and 1.0 g muriate of potash (KCl). An additional 2.5 g (NH_4_)_2_SO_4_ was top-dressed at 30 days after transplanting. One plant per pot was grown under fully flooded conditions and was grown in the greenhouse conditions before imposing stress at the targeted stages (panicle initiation until physiological maturity). The 209 genotypes were grouped into five batches based on flowering time information from Kadam et al.^38^. The five batches were stagger sown with a 5-day interval between each batch (Fig. 1a) and transplanted with the same intervals to ensure best possible synchrony in panicle initiation and flowering. Representative plants for each batch were monitored and used to determine the panicle initiation stage by destructive inspection^39^.

### Walk-in chambers and HNT stress imposition

Six walk-in chambers, each with a total area of 10.6 m^2^ (3.3 m [length] × 3.2 m [width] × 2.7 m [height]) were used in the study (Supplementary Fig. S1a). Each chamber was fitted with six independent units of 1 kW high-intensity discharge lamps, providing a photosynthetic photon flux density (PPFD) of ~ 650 µmol m^−2^ s^−1^ at 1.2 m from the base of the chamber. A photoperiod of 11 h (13 h of dark) and a constant relative humidity of ~ 70% was maintained throughout the stress period. The HNT temperature in the chambers was maintained at 29 °C for 11 h during the night (18:30 to 05:30 h), with an optimum of 31 °C during the day (06:30 to 17:30 h) starting from panicle initiation until physiological maturity. A linear change in temperature from day to night and vice versa was programmed between 17:30 and 18:30 h; and 05:30 and 06:30 h. MINCERs (Micrometeorological Instrument for Near Canopy Environment of Rice, developed by the National Institute of Agrobiological Sciences, Japan) was placed in the middle of the chambers with temperature sensors above the plant canopy. MINCERs equipped with data loggers (RX-350TH, As One Co., Osaka, Japan) were used to record ambient air temperature and relative humidity at 15 min intervals both in the greenhouse and in the walk-in chambers. Three replicate plants for each genotype were transferred into three different HNT chambers at panicle initiation. Simultaneously, a similar number of plants (three plants per genotype) were maintained under greenhouse conditions throughout the crop growth cycle and considered as absolute controls. In the Exp. 1, temperatures in control (greenhouse) and HNT treatments (walk-in chambers) were 26.6 °C (standard deviation, SD = 0.4) and 29.3 °C (SD = 0.61 °C), respectively. The relative humidity during the treatment period was 82.2% (SD = 1.2%) in the control treatment, 80.5% (SD = 1.3%) in the HNT treatment (controlled environment experiment; Fig. 1b). Amplitude (temperature difference between day and night) during the HNT stress treatment was 2.3 °C higher during the night (Fig. 1b).

## Experiment 2 (Field-based heat tents)

### Crop management

This experiment was carried out during the dry season (January to May of 2016) at the Experimental Farm, IRRI. The pre-germinated seeds of 209 cultivars were staggered sown [similar to Exp. 1] in a seeding tray filled with the same clay loam farm soil and fourteen-day-old seedlings were hand transplanted at a spacing of 20 × 20 cm. Each row had 15 plants (rows were not replicated, due to limitation in space), in an augmented design. All the genotypes were grown under fully flooded conditions, with about 2 cm of standing water at transplanting and about 5 cm from crop establishment until physiological maturity. Nitrogen (N), phosphorus (P), potassium (K) [150:30:40, N:P:K kg ha^−1^] and micronutrient zinc were applied as urea (150 kg ha^−1^), single superphosphate (30 kg ha^−1^) and muriate of potash (40 kg ha^−1^) along with zinc sulfate heptahydrate (5 kg ha^−1^). All the fertilizers were incorporated into the plots as a basal dose (1 day before transplanting) except for urea, which was applied in four splits of 3:2:3:2 at basal, mid-tillering, panicle initiation, and heading stage, respectively. All the agronomic and crop management practices were followed, similar to Shi et al.^11^ and Bahuguna et al.^13^.

### HNT stress using field-based tents

All genotypes were exposed to two-night temperature treatments i.e., control night (CNT; 24 °C) and high night temperatures (HNT; 29 °C) with a common day-time temperature across both treatments. Plants were exposed to 24 °C (CNT) or 29 °C (HNT) night temperature by using field-based tents between (18:00 to 06:00 h) and during the day-time (06:00 to18:00 h) the tents were open, exposing the plants to natural conditions [Supplementary Fig. S1]. To start the HNT stress, the sides of the tents were manually rolled down at 18:00 h every evening and re-opened at 6:00 h in the morning, exposing the plants to12 h of photoperiod. Each tent was outfitted with an air conditioner, which was programmed to automatically regulate control (24 °C) and stress (29 °C) temperatures at night. Heat tents were equipped with temperature and relative humidity sensors and were connected to data loggers (HOBO, Onset Computer Corp., Bourne, MA). These were fixed in each tent above the canopy to record environmental conditions at 15-min intervals. Average night (18:00–06:00 h) temperature during stress treatment (from panicle initiation until maturity) was 24.3 °C (SD = 0.7) for the control and 28.3 °C (SD = 0.53) with HNT in Exp. 2 (Field experiment; Fig. 1b). Amplitude (the difference between day and night) temperatures during the stress treatment was 5.8 °C (Fig. 1b).

### Phenotypes and statistical analysis

At physiological maturity, about three (Exp. 1) to twelve plants (Exp. 2) were harvested separately to determine the impact of HNT on the grain yield per plant (YPP) and 100-grain weight (HWT), harvest index (HI), and chalkiness (CHALK). Panicles were hand-threshed to estimate grain yield per plant and 100-grain weight. The harvest index was calculated as the ratio of grain yield to the plant's total dry matter. In Exp. 2, representative samples from each genotype were analyzed for grain size and grain chalkiness at the Grain Quality and Nutrition Center, IRRI, Philippines, following the ISO 17025 certified protocols of the IRRI GQNSL (http://www.knowledgebank.irri.org/ricebreedingcourse/bodydefault.htm#Grain_quality.htm). All phenotypic traits were subjected to statistical analysis to detect the effects of genotype and treatment using the library ("agricolae") RStudio 3.6.1 (https://rstudio.com/). Least square difference (LSD) was used to compare the mean values differences between control and HNT. Pearson’s correlation coefficient was carried out between all traits using the library ("corrplot") in RStudio 3.6.1. The relative performance of a genotype under stress was determined using the following equation.

$$Relative\, value = \frac{{\text{Absolute trait value at high night temperature }}}{{\text{Absolute trait value at control night temperature }}}$$

### Genome-wide association analysis (GWAS)

One hundred and seventy-three genotypes in Exp. 1 (panicles did not emerge in 36 genotypes under HNT), and 206 genotypes in Exp. 2 had complete phenotypic data. However, the genotypic data for twenty genotypes were missing, and hence only those genotypes that had both genotypic and phenotypic data (158 genotypes in Exp. 1 and 188 in Exp. 2) were used for GWAS analysis. GWAS was performed to estimate the association between marker-trait using the Genomic Association and Prediction Integrated Tool^40^. A mixed-model approach was implemented in the Compressed Mixed-Model Association (CMLM), which controls the familial relatedness and population structure and kinship (as a random effect to control population structure) to minimize false-positive associations^41^. A total of 45, 200 single nucleotide polymorphisms (SNPs; minor allele frequency > 0.05) that were generated under the Global Rice Phenotyping Network (http://ricephenonetwork.irri.org) were used for performing GWAS^38,42,43^. Estimated absolute phenotypic or relative values along with PC and Kinship were used to discover marker-trait associations (MTAs). The Bonferroni correction had a more stringent threshold; therefore it did not result in many significant associations, so we have used a significant (*–log(p)* = *3.5*) threshold to discover MTAs similar to several previous studies^42,44^. Manhattan plots were constructed using the library ("qqman") in RStudio 3.6.1. (https://cran.r-project.org/web/packages/qqman/vignettes/qqman.html). The SNP with the lowest *p-*value within 100 kb is considered as a candidate marker-trait association (cMTA) to represent that locus. Thus, the SNP with minimum *p‐value* for each locus was defined as cMTA. Then we compared cMTAs/peak SNP to detect shared loci and unique loci across treatments and experiments. Candidate genes were searched in ± 50 kb from the cMTAs in rap-db on IRGSP-1.0 (http://rapdb.dna.affrc.go.jp/).

### Prioritizing gene candidates

Part of this study was aimed to reduce the inherent bias in selecting candidate genes based on existing literature by incorporating alternate ways to identify the top genes of interest^45^. To the 638 candidate genes identified in the 100-kb window surrounding the unique 51 cMTAs, three potential gene prioritization approaches were applied: (1) Comparison to genes previously identified as responsive to HNT via differential expression analysis, (2) identification of genes highly connected to transcriptional regulators through a gene regulatory network, and (3) genes orthologous to genes previously identified as associated with traits through QTG-Finder2^46^. These three comparisons integrate data from different experimental approaches to narrow down a list of potential candidate genes.

## Prioritization based on differential expression analysis

This approach used altered expression in response to HNT as a filter to prioritize candidate genes. A prior experiment performed RNA-Sequencing analysis on the *O. sativa* genotype IR64, grown under similar experimental conditions at IRRI, Philippines (data accessible at NCBI GEO database^37^, accession GSE159073). RNA-Seq data from this prior time course study from flag-leaf tissue was normalized, mapped, and expression count per gene obtained as described in Desai et al.^37^. Only 354 genes, 223 in the genes identified from the absolute analysis and 131 from the relative analysis, were in the candidate gene list from trait loci and had detectible expression levels in the initial transcriptomic analysis. The expression data were analyzed using the R package edgeR^47^ to identify genes differentially expressed at any time point. Candidate genes were prioritized when identified as significantly differentially expressed (FDR adjusted *p-*value < 0.05) for any of the comparisons between HNT and control.

### Prioritization based on connectedness in a gene regulatory network

We used a previously published gene regulatory network created using expression data from different rice developmental stages^37^. The connectedness of transcriptional regulators and their target genes in the network was visualized and examined using the R package networkD3^48^. In this network, the confidence that a transcription factor interacts with a target gene is based on the strength of the association. Interaction scores ranged from 0 (the transcription factor is unlikely to interact with the target) to 871 (a high-confidence interaction). The full network was subset to include only interaction scores greater than or equal 200 to focus on transcription factors and target genes with strong associations. Only 0.3% of the interactions passed this stringent filter, leaving 45,879 interactions between transcription factors and target genes. The 638 annotated genes identified in the GWAS were compared to these networks. The gene candidates were evaluated based on the strength and number of interactions it had with transcriptional regulators.

### Orthologous genes

The last approach used to prioritize candidate genes used the relationship to known causal genes to determine the best candidate genes. Lin et al.^46^ developed a machine learning approach to prioritizing association-identified loci called QTG-Finder. QTG-Finder takes a list of QTL loci and their associated potential candidate genes from a locus (or loci) and ranks each gene in order from the most to least likely to be a causal gene for each respective locus. This approach has been tested and works well in *O. sativa* and *A. thaliana*^46^. This has recently been extended in QTG-Finder2 to use orthologous genes to identify causal genes in Setaria and Sorghum based on rice and Arabidopsis studies^46^. The list of candidate genes from the GWAS study was submitted to QTG-Finder2 to rank the genes per locus. The genes were translated from RAP_ID to MSU_ID format to be compatible with the software. This reduced the total to 523 genes, 333 candidate genes from 34 absolute loci and 190 genes from the 20 relative loci. QTG-Finder2 could not be run on two loci due to an error we could not resolve. Of the remaining loci, the top-ranked genes for each locus were considered to be a prioritized candidate gene. Top-ranked genes were the top two genes for each locus ranked by QTG-Finder2.

## Results

### Phenotypic diversity and correlations among traits

**Figure 2:**
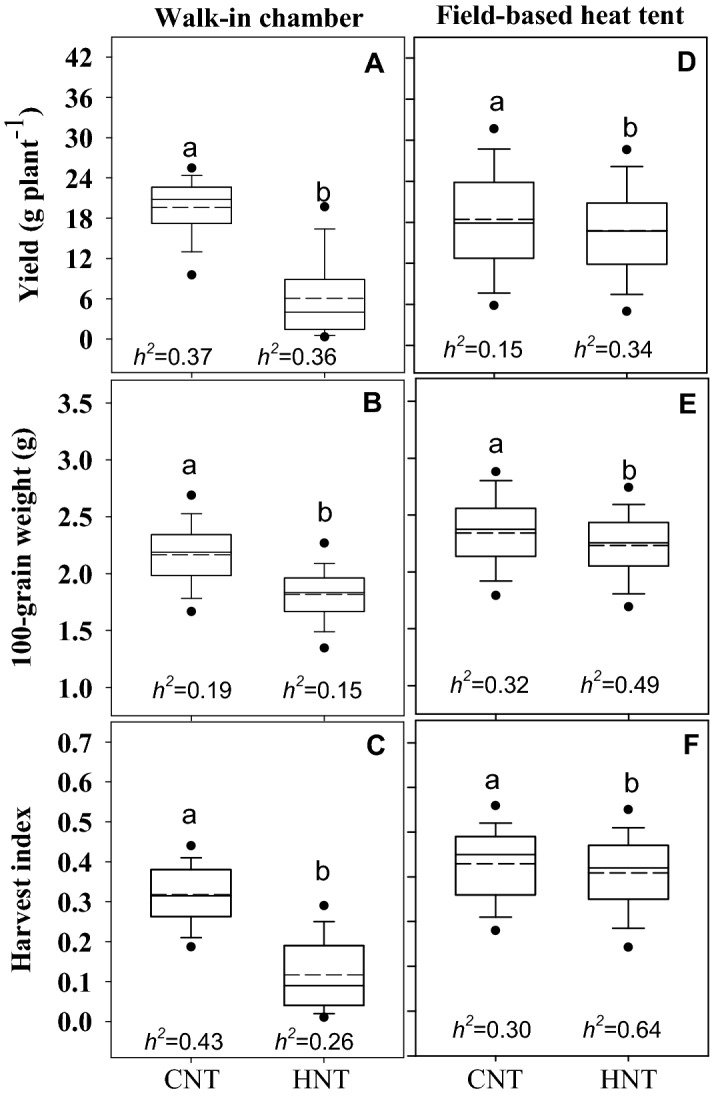
Boxplots showing the phenotypic responses of traits (**A**, **D** yield; **B**, **E** 100-seed weight; **C**, **F** harvest index) under control and high night temperature stress in Exp. 1 (n = 173) and Exp. 2 (n = 206). In Exp. 1 (walk-in chambers; **A**-**C**), temperature treatments included control night temperature (CNT) and high night temperature (HNT) starting from panicle initiation to physiological maturity. In Exp. 2 (**D**-**F**), genotypes were exposed to control night (CNT) and high night temperature (HNT) with common day-time ambient temperature across treatments. The solid and dotted line represents the median and means of the data. The closed circles at the boundary show outliers at 90th percentile. The marker-based heritability (*h*^*2*^) was obtained in the Genomic Association and Prediction Integrated Tool. Different letters indicate statistically significant least square difference (LSD) for treatments at the level of *p* < 0.05.

All the traits examined recorded significant differences (*p* ≤ 0.05) under control and HNT conditions both in the walk-in chamber (Exp. 1, Fig. 2a–c) and field experiments (Exp. 2, Fig. 2d–f). A wide range in phenotypic variability was observed in the diversity panel for yield per plant, 100-seed weight, and harvest index (Fig. 2). Marker-based heritability values were low to intermediate, ranging from 0.15 (100-seed weight in Exp. 1 under HNT and grain yield in Exp. 2 under control) to 0.64 (chalkiness under HNT in Exp. 2). Genotypes under HNT stress resulted in significantly lower phenotypic values compared to control (Fig. 2), with reductions ranging from 4.7% (100-seed weight in Exp. 2, Fig. 2e) to 69% (grain yield in Exp. 1, Fig. 2a). Yield (69%), 100-seed weight (16%), and harvest index (63.3%) decreased more significantly under HNT compared to control, in the controlled-environment study (Exp. 1, Fig. 2a–c). An increase in night-time temperature from 24 to 29 °C in the field-based heat tents resulted in a two-fold increase in chalkiness.

**Figure 3:**
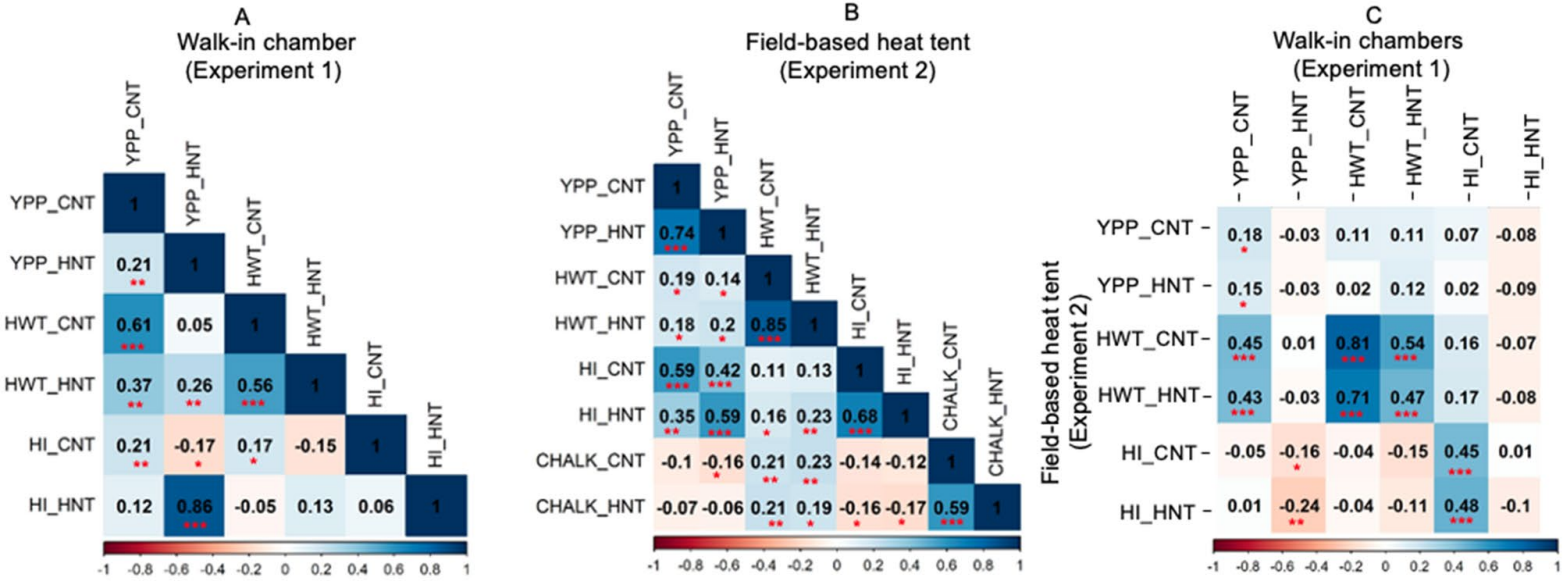
Pearson’s correlation coefficients between the traits in walk-in chambers (173 genotypes in Exp. 1; **A**) field-based heat tents (206 genotypes in Exp. 2; **B**) and between experiments (Exp. 1 vs. Exp. 2, **C**). In experiment 1 (walk-in chambers), temperature treatments included control night temperature (CNT) and high night temperature (HNT), and in experiment 2, two different night temperature treatments i.e., control night (CNT) and high night temperature (HNT) were imposed with common day-time ambient temperature across treatments. YPP- grain yield (g per plant); HWT-100-seed weight (g); *HI* Harvest index and *CHALK* chalkiness (%). *p < 0.05, **p < 0.01, ***p < 0.001 indicate significant correlations among traits.

**Figure 4:**
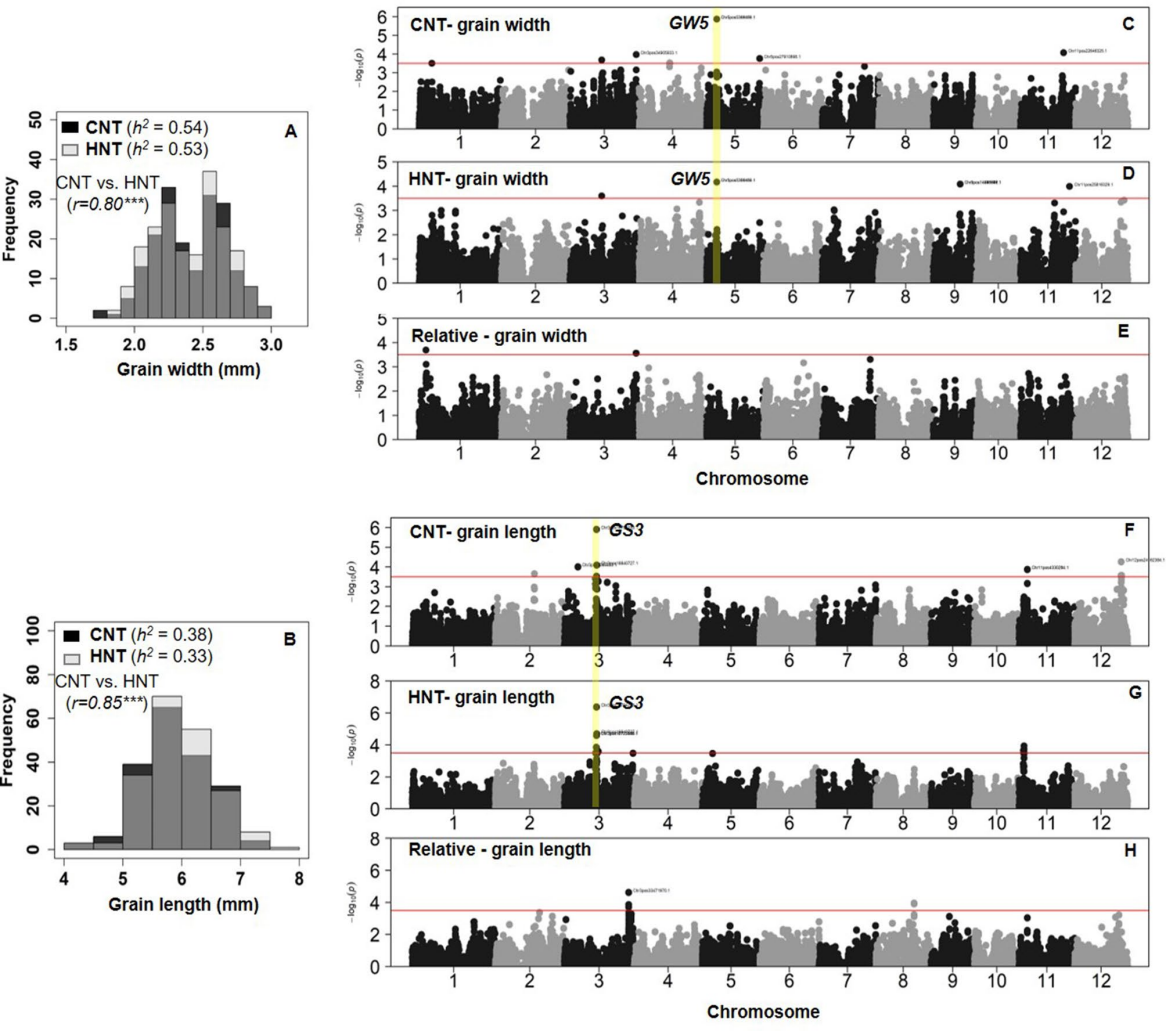
Rice grain width (mm, **A**) and grain length (mm, **B**) in response to control night (CNT; 24 °C, covered tents) and high night temperatures (HNT; 29 °C, covered and heated) under common day-time temperature under field conditions (Exp. 2; Supplementary Fig. S1). Manhattan plots of genome-wide association results for rice mature grain width (**C**–**E**) and length (**F**–**H**) in the study. The marker-based heritability (*h*^*2*^) was obtained in the Genomic Association and Prediction Integrated Tool. The previously characterized major genes controlling grain shape traits (yellow bar) such as the grain width (*GW5*, **C**,**D**) on chromosome 5 and grain length (*GS3*, **F**,**G**) genes on chromosome 3 are labeled. *r*—Pearson’s correlation coefficient between treatments. ***p < 0.001 indicate significant correlation of traits between CNT and HNT.

Yield per plant (YPP, *r* = 0.21, *p* < 0.01) and 100-seed weight (HWT, *r* = 0.56,* p* < 0.001) were correlated significantly between the two treatments in Exp. 1 (Fig. 3a). Similarly, all the measured traits (YPP, *r* = 0.74; HWT, *r* = 0.85; and HI, *r* = 0.68, *p* < 0.001), including chalkiness (CHALK, *r* = 0.59, *p* < 0.001) were strong and positively correlated between the two treatments in Exp. 2 (Fig. 3b). The yield under control condition (in Exp. 1) showed positive correlations with yield and 100-seed weight, under both treatments with Exp. 2 (Fig. 3c). Strong significantly positive correlations were observed between Exp. 1 and Exp. 2 across treatments for the 100-grain weight (Fig. 3c). Yield under HNT in Exp. 1 was negatively correlated (*r* = *− *0.24,* p* < 0.01) with the harvest index in Exp. 2 (Fig. 3c). Grain width (*r* = 0.80, *p* < 0.001*;* Fig. 4a) and grain length (*r* = 0.85, *p* < 0.001) were strongly correlated across treatments in Exp. 2 (Fig. 4b). Nonetheless, the correlations between the grain width and length were consistent for both absolute (*r* = − 0.65, *p* < 0.001) under HNT and relative value (*r* = − 0.50, *p* < 0.001).

## Genome-wide association study

### Experiment and treatment specific MTAs

We identified genomic regions associated with different traits under control (representing controlled non-stress night temperature), HNT (representing high night temperature) and relative (representing genotype response as a ratio of HNT to CNT), by CMLM (Supplementary Figs. S2 and S3). A summary of GWAS results is given in Table 1, and the list of peak SNPs or candidate MTAs (cMTAs) for each locus are presented in Table 2. Significant MTAs varied between traits (absolute and relative values), from a minimum of five to a maximum of 25 (Table 1). The phenotypic variance (R^2^) explained by each marker ranged from 0.07 (relative yield in Exp. 2) to 0.37 for chalkiness (in Exp. 2) under HNT (Table 1). A total of 75 MTAs were associated with measured phenotypic traits under HNT, of which 24 were highly significant (*p* ≤ 7E−06 to 9E−05), 20 were significant at *p value* = 1E−04, and the rest met a suggestive threshold (*p* ≤ 2E−04 to 6E−04) (Supplementary Table S1). Sixty-one MTAs were detected (Supplementary Table S1) for relative values from two experiments (Supplementary Figs. S2 and S3).

**Table 1:** Summary of significant marker-trait associations (MTAs) with yield, 100-seed weight, harvest index, and chalkiness traits in experiment 1 (walk-in chamber study) and experiment 2 (field-based heat tent study) in rice.

Trait	Treatment	Chr	MTAs	P value		R^2^ of model with SNP	
				Minimum	Maximum	Minimum	Maximum
**Walk-in chamber study**							
Yield (g plant^−1^)	CNT	1,2	5	5E−05	2E−04	0.20	0.21
	HNT	2,11	15	7E−06	5E−05	0.32	0.34
	Relative	1,2	14	4E−05	8E−05	0.27	0.27
100-seed weight (g)	CNT	2,7,9	5	1E−05	2E−04	0.15	0.18
	HNT	2,8	5	8E−05	3E−04	0.25	0.26
	Relative	3,8	3	2E−05	9E−05	0.15	0.17
Harvest index	CNT	1,4	6	2E−04	2E−04	0.29	0.29
	HNT	2,4,11	25	1E−04	4E−04	0.23	0.25
	Relative	2,11	19	4E−05	2E−04	0.28	0.30
**Field-based heat tent study**							
Yield (g plant^−1^)	CNT	2,4	5	1E−04	2E−04	0.09	0.10
	HNT	2,8	5	1E−04	2E−04	0.15	0.16
	Relative	5,7	2	2E−04	5E−04	0.07	0.08
100-seed weight (g)	CNT	2	12	4E−05	8E−05	0.15	0.16
	HNT	3,4	5	2E−05	9E−05	0.23	0.24
	Relative	4,5,8	3	2E−04	3E−04	0.30	0.30
Harvest index	CNT	1,3,11,12	18	5E−05	2E−04	0.15	0.17
	HNT	1,7,11	11	3E−05	6E−04	0.16	0.19
	Relative	6,7	3	2E−05	2E−04	0.09	0.12
Chalkiness	CNT	3,9,11	16	1E−05	8E−05	0.16	0.18
	HNT	1,2,3,4	9	9E−05	2E−04	0.36	0.37
	Relative	2,6,8,11	13	6E−07	1E−04	0.31	0.37

The list of significant MTAs and Manhattan plots are presented in Supplementary Table S1, Figs. S3, and S4, respectively.

*CNT* control, *HNT* high night temperature treatments, *Chr* chromosome, *Relative* high night temperature/control night temperature.

**Table 2:** List of candidate MTAs identified under control night (CNT) and high night temperature (HNT) in walk-in chambers (Experiment 1- E1) and field-based heat tents (Experiment 2- E2).

Trait	Locus	Experiment	Treatment	SNP name	P value	Allele	AE	Favorable allele	No. of genes in ± 50 kb
Yield per plant (YPP)	Q1	E1	CNT	Chr1pos6542949.1	8E−05	G:A	5.92	A	16
	Q2	E1	CNT	Chr2pos31467953.1	5E−05	G:A	− 3.24	G	14
	Q3	E2	CNT	Chr2pos31622043.1	1E−04	G:T	− 5.29	G	13
	Q4	E2	CNT	Chr4pos31966920.1	2E−04	G:C	4.61	C	16
	Q5	E1	HNT	Chr1pos22255654.1	7E−06	G:C	12.02	C	3
	Q6	E1	HNT	Chr2pos34468097.1	4E−05	T:G	6.32	G	15
	Q7	E1	HNT	Chr11pos23922165.1	4E−05	T:C	9.21	C	14
	Q8	E2	HNT	Chr2pos31666003.1	1E−04	T:G	− 4.53	T	20
	Q9	E2	HNT	Chr8pos1149355.1	1E−04	C:T	4.63	T	12
	YPP_Rel	E1	Relative	Chr1pos22255654.1	4E−05	G:C	0.54	C	4
	YPP_Rel	E1	Relative	Chr2pos34468097.1	6E−05	T:G	0.30	G	14
	YPP_Rel	E2	Relative	Chr7pos23086735.1	2E−04	C:A	0.32	A	17
100− seed weight (HWT)	Q10	E1	CNT	Chr2pos484762.1	1E−05	C:G	− 0.36	C	15
	Q11	E1	CNT	Chr7pos20225421.1	2E−04	T:C	− 0.14	T	19
	Q12	E2	CNT	Chr2pos31959843.1	4E−05	A:G	− 0.22	A	17
	Q13	E1	HNT	Chr2pos4831761.1	8E−05	C:A	− 0.15	C	19
	Q14	E2	HNT	Chr3pos6079159.1	2E−05	C:T	− 0.20	C	11
	Q15	E2	HNT	Chr4pos4550145.1	7E−05	C:T	− 0.11	C	7
	HWT_Rel	E1	Relative	Chr3pos15905968.1	2E−05	G:A	0.17	A	6
	HWT_Rel	E1	Relative	Chr8pos4667697.1	9E−05	G:C	0.10	C	11
	HWT_Rel	E2	Relative	Chr4pos33782238.1	2E−04	G:A	− 0.06	G	19
	HWT_Rel	E2	Relative	Chr5pos29593994.1	3E−04	A:G	− 0.07	A	20
	HWT_Rel	E2	Relative	Chr8pos25495868.1	2E−04	G:A	− 0.14	G	9
Harvest index (HI)	Q16	E1	CNT	Chr1pos6652979.1	2E−04	T:G	− 0.08	T	15
	Q17	E1	CNT	Chr4pos15947167.1	2E−04	A:G	0.07	G	4
	Q18	E2	CNT	Chr1pos29483935.1	5E−05	T:A	− 0.10	T	17
	Q19	E2	CNT	Chr1pos30060165.1	1E−04	C:T	− 0.08	C	23
	Q20	E2	CNT	Chr11pos23898551.1	6E−05	C:T	− 0.13	C	2
	Q21	E2	CNT	Chr11pos24244335.1	1E−04	A:G	− 0.13	A	11
	Q22	E1	HNT	Chr2pos25528306.1	2E−04	G:T	0.14	T	14
	Q23	E1	HNT	Chr2pos34468097.1	1E−04	T:G	0.07	G	15
	Q24	E1	HNT	Chr4pos28033149.1	2E−04	C:T	0.10	T	19
	Q25	E1	HNT	Chr11pos3631830.1	4E−04	T:C	0.09	C	10
	Q26	E2	HNT	Chr1pos4312404.1	3E−05	C:A	− 0.12	C	12
	Q27	E2	HNT	Chr11pos17764785.1	3E−04	A:C	0.06	C	5
	HI_Rel	E1	Relative	Chr2pos23789929.1	4E−05	G:T	0.45	T	14
	HI_Rel	E1	Relative	Chr11pos3631830.1	2E−04	T:C	0.34	C	10
	HI_Rel	E2	Relative	Chr6pos15730222.1	2E−04	T:C	− 0.20	T	9
	HI_Rel	E2	Relative	Chr7pos6689342.1	2E−05	G:A	− 0.23	G	3
Chalkiness (CHALK)	Q28	E2	CNT	Chr3pos15967569.1	4E−05	G:T	13.42	G	12
	Q29	E2	CNT	Chr9pos14091989.1	4E−05	G:A	16.62	G	10
	Q30	E2	CNT	Chr11pos22646338.1	1E−05	C:G	14.12	C	–
	Q31	E2	HNT	Chr1pos39165647.1	1E−04	A:G	39.61	A	14
	Q32	E2	HNT	Chr2pos33430148.1	1E−04	T:C	27.26	T	15
	Q33	E2	HNT	Chr3pos1662112.1	1E−04	A:C	− 17.80	C	16
	Q34	E2	HNT	Chr4pos20331507.1	9E−05	T:C	24.36	T	16
	CHALK_Rel	E2	Relative	Chr2pos18751285.1	6E−07	G:A	10.67	A	6
	CHALK_Rel	E2	Relative	Chr6pos909702.1	7E−05	T:G	8.39	G	18
	CHALK_Rel	E2	Relative	Chr8pos20389531.1	2E−05	C:G	− 8.75	C	10
	CHALK_Rel	E2	Relative	Chr11pos1113438.1	1E−05	G:A	9.19	A	8
Grain width (GW)	GW5	E2	CNT	Chr5pos5366489.1	1E−06	G:A	0.39	A	6
	GW5	E2	HNT	Chr5pos5366489.1	7E−05	G:A	0.30	A	6
	GW_Rel	E2	Relative	Chr1pos3609856.1	2E−04	A:G	0.042	G	10
	GW_Rel	E2	Relative	Chr7pos25293594.1	5E−04	A:G	0.053	G	20
Grain length (GL)	GL3	E2	CNT	Chr3pos16794259.1	4E−06	A:T	0.32	T	7
	GL3	E2	HNT	Chr3pos16794259.1	4E−07	A:T	0.77	T	7
	GL_Rel	E2	Relative	Chr3pos33471970.1	2E−05	C:T	0.072	T	21

Allelic effect with respect to minor allele (AE) = (average phenotypic value of accessions with the minor allele-average phenotypic value of accessions carrying the major allele). Annotated genes housed in ± 50 kb of the cMTAs/locus identified in the study. Rel- relative (high night temperature/control night temperature).

### Candidate MTAs across treatments

A total of 18, 20 and 19 cMTAs (SNP with a minimum *p‐value* for each locus) were identified for the traits under control, HNT and for relative values, respectively (Table 2). Twenty candidates MTAs were identified for the yield and quality-related traits i.e., yield (5), 100-seed weight (3), harvest index (6), chalkiness (4) and one each for grain width and grain length under HNT across experiments (Table 2). We also noticed that the identified genetic loci for grain width (Chr5pos5366489.1) and grain length (Chr3pos16794259.1) co-localized on the well-characterized, previously identified genes across treatments (Fig. 4). Three co-localizing cMTAs were detected between relative and absolute values for grain yield (Chr1pos22255654.1, Chr2pos34468097.1) and harvest index (Chr11pos3631830.1).

We detected three (Q5–Q7) and two (Q8–Q9) loci for grain yield under HNT, in Exp.1 and Exp.2, respectively. Minor alleles of five grain yield loci (Q5–Q9) revealed small (− 4.53 g) to medium (12.02 g) allelic effects in HNT conditions (Table 2). The allelic variation explained by the SNP markers of loci Q5-Q7 (i.e., Chr-1pos22255654.1, Chr2pos34468097.1 and Chr11pos23922165.1) showed that accessions carrying alleles ‘C, G and C’ had a higher grain yield under HNT (in Exp. 1) compared to accessions carrying ‘G, T and T’ alleles, respectively (Table 2). In Exp. 2, the minor allele of Q8 (Chr2pos31666003.1) had a negative effect on yield, while the minor allele of Q9 (Chr8pos1149355.1) had a positive impact on yield (Table 2). Three loci (Q2, Q3, and Q8; different SNPs but falls within the same LD block) on chromosome 2 clustered within 0.49 Mb region and were commonly associated with yield across treatments (Table 2). Three cMTAs were identified for relative grain yield (Table 2). Of the three cMTAs, two SNPs (Chr1pos22255654.1 and Chr2pos34468097.1) were shared between the grain yield under HNT and relative grain yield in Exp. 1 (Table 2, Supplementary Fig. S2).

Test weight (HWT; 100-seed weight) is a key yield component and is shown to be highly positively correlated with yield (Fig. 3) and significantly affected by HNT. We identified three loci (Q10–Q12) for 100-seed weight under control. The minor alleles of all three loci had a negative effect on 100-seed weight in both Exp. 1 and Exp. 2 (Table 2). Three cMTAs (one on chromosome 2 [Q13] in Exp. 1; one on chromosome 3 [Q14], and 4 [Q15] in Exp. 2) were associated with the 100-seed weight under HNT (Table 2). Similarly, the minor alleles of all three loci (Q13, Q14, and Q15) had a negative effect on 100-seed weight under HNT. Based on the relative 100-seed weight, five cMTAs were detected (2 in Exp. 1 and 3 in Exp. 2). Of the five cMTAs, minor alleles of three loci (Chr4pos33782238.1-‘G’; Chr5pos29593994.1-‘A’; Chr8pos25495868.1-‘G’) from Exp. 2 had negative effects on relative 100-seed weight (Table 2).

Harvest index has been used as a potential proxy for partitioning capacity between source and sink both under non-stress and stress conditions. Six loci (Q16-Q21) were detected for harvest index under control. Minor alleles of the loci associated with harvest index affecting the trait negatively under control (Table 2), except locus Q17 (Chr4pos15947167.1) (Table 2). We detected four and two genetic loci for harvest index in Exp. 1 (Q22–Q25) and Exp. 2 (Q26 and Q27) under HNT, respectively. Four markers, Chr2pos23789929.1, Chr6pos15730222.1, Chr7pos6689342.1, and Chr11pos3631830.1, were associated with relative harvest index across experiments (Table 2). For harvest index, cMTAs under HNT were located on chromosomes 1, 2, 4, and 11, and the most significant favorable allele associated with the Chr2pos25528306.1 (Q22, T) positively influenced the trait by 14% (*p* = 2E−04) in Exp. 1 and Chr1pos4312404.1 (Q26, C) influenced the same trait by 12% (*p* = 3E−05) in Exp. 2, under HNT (Table 2). In Exp. 2 the minor allele of Q26 had a negative impact on the harvest index, while the minor allele at Q27 affected the same trait positively under HNT (Table 2). Genomic regions (Q22–Q27) associated with harvest index under HNT were in close proximity to the region of genes controlling plant nutrient status (phloem sucrose transport, nitrogen metabolism, photoperiodic control of flowering, osmotic stress response, and lipid homeostasis) (Supplementary Tables S2 and S3). In Exp. 1, GWAS identified a key region on chromosome 2 (34.46 Mb) that was commonly associated with harvest index (Q23) and yield (Q6) under HNT (Table 2). Out of four cMTAs detected across experiments for relative harvest index, one candidate SNP (Chr11pos3631830.1) was shared between absolute and relative harvest index in Exp. 1 (Table 2).

Chalkiness is one of the primary factors determining rice milling and appearance qualities, both under control and stress conditions^11^. Eleven cMTAs (3 in control, 4 in HNT and 4 for relative) were identified for chalkiness in Exp. 2 (Table 2, Supplementary Fig. S3). Under control conditions, GWAS identified locus (Q30, Chr11pos22646338.1) on chromosome 11 (22.6 Mb) that was highly associated with chalkiness (Supplementary Fig. S3b), but not for HNT (Supplementary Fig. S3c). The minor allele of the lead SNP (Chr3pos1662112.1) underlying the peak Q33 had a negative effect (− 17.8%) on chalkiness (Table 2). Another cMTA (Q31) for chalkiness had the highest positive allele effect (39.6%) under HNT, detected on chromosome 1. A minor locus (Q34) on chromosome 4 (Chr4pos20331507.1) induced chalkiness (effect = 24.3%) under HNT. Also, one genomic region on chromosome 4 (19.9–20.3 Mb) for chalkiness was found to be co-localized both under control and HNT conditions (Supplementary Fig. S3b, c). Among the identified loci for relative chalkiness in Exp. 2, a SNP (Chr2pos18751285.1) on chromosome 2 was strongly (*p* = 6E−07) associated with relative chalkiness (Table 2). The Chr2pos18751285.1 SNP marker's minor allele had a positive effect on relative chalkiness (Table 2).

Grain shape/size (GS) is usually evaluated by grain width (GW)and grain length (GL), which are the key grain quality traits that influence the rice consumer preferences. Interestingly, a highly significant association (*p* = 1E−06 and *p* = 7E−05) was detected for grain width (5.3 Mb) on chromosome 5 across treatments (CNT, and HNT, respectively) (Fig. 4c,d), which is in the region of a well-characterized gene, *qSW5/GW5* (encoding a calmodulin-binding motif family protein) that regulates grain width in rice^49,50^. No significant common loci were detected for grain width between absolute and relative values (Fig. 4e). Similar to absolute grain width, the SNP (Chr3pos16794259.1) associated with grain size (*GS3*)^51,52^, was detected as most significantly associated locus with absolute grain length across treatments (Fig. 4f,g). No loci were shared between the absolute and relative grain length. However, one SNP (Chr3pos33471970.1) on chromosome 3 was significantly associated with relative grain length but not co-located with the *GS3* locus (Fig. 4h).

Overall, we found two prominent GWAS loci peaks on chromosome 2 (region 1: 31.4–31.9 Mb and region 2: 34.6–34.7) influencing grain yield and harvest index in rice (Supplementary Fig. S2). Region 1 (31.4–31.9 Mb) is associated with Q2, Q3, Q8, and Q12 that control yield and 100-seed weight either under control or HNT (Table 1, Supplementary Fig. S2). Similarly, region 2 (34.5 on chromosome 2; Q6 and Q23) commonly associated with absolute grain yield and harvest index under HNT and relative grain yield and relative harvest index in Exp. 1 (Table 2, Supplementary Table S1). A SNP marker associated with Chr1pos22255654.1 (Q5) co-located with the relative grain yield in Exp. 2 (Table 2).

### Prioritizing gene candidates

An *in-silico* search for all annotated genes in a 100-kb window surrounding the unique 51 candidate SNPs yielded 638 (396 for absolute, 209 for relative and 33 shared between absolute and relative) annotated genes, ranging between 2 (Q20) and 23 (Q19) genes (Table 2, Supplementary Table S2). We found that these genes encode several classes of protein with known or predicted functions in abiotic and grain development pathways, including helix-loop-helix DNA-binding domain-containing proteins, similar to starch synthase, GRAS transcription factor domain-containing protein, a calcium-binding protein, mitogen-activated protein kinase 4, ROS scavenging, sugar signaling and metabolism, and auxin-responsive protein (Supplemental Table S2). Further, a search for characterized genes within candidate loci yielded 127 functional candidate genes (http://funricegenes.ncpgr.cn/) with a range of 1–17 candidate genes per locus (Supplementary Table S3).

A common approach is prioritizing candidates for each locus based on functional roles from prior literature and coding variants. However, the negative effects of HNT manifest through different mechanisms than HDT, and little is known about the underlying genes driving the genetic architecture of these traits under HNT. Therefore, previous literature may not cover the genes involved in HNT response regulation. Moreover, since non-coding variants can play critical roles in trait expression, prioritizing coding variants ignores significant non-coding regulatory variants. Therefore, three independent approaches were applied to prioritize candidate causal genes driving the traits' genetic architecture under both control and HNT conditions. The annotated genes in the 100-kb window surrounding each locus were examined for overlap in three ways: (1) with genes differentially expressed in response to HNT, (2) strength of association in a gene regulatory network, and (3) genes orthologous to prior causal genes. This approach reduces the bias by prioritizing candidate genes based on other data sets.

**Figure 5:**
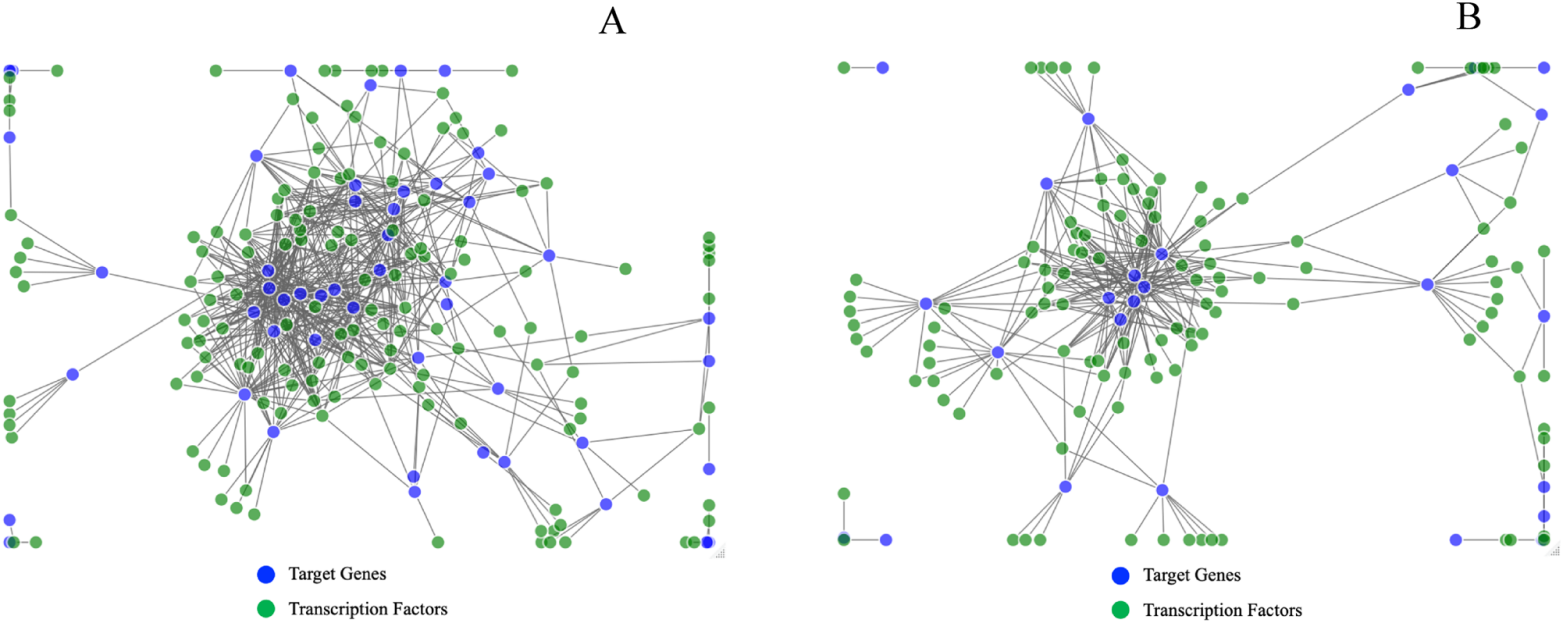
Gene regulatory networks are made up of top candidate genes from the GWAS of absolute (**A**) and relative trait values (**B**) and candidate genes with strong transcription factor interaction levels (> 200). A list of transcription factors for each candidate is given in Supplementary Table S5.

The first approach, which examined if candidate genes were differentially expressed in HNT, found 34 significant candidate genes, 22 of the genes from loci identified in the absolute analysis and 12 from the relative loci. The gene regulatory network approach prioritized 76 genes based on their interaction with transcription regulators, 50 from the absolute candidates and 26 from the relative candidates (Fig. 5a,b). The final method, which prioritized candidate genes based on their orthogonality to known causal genes, ranked 97 genes as top priority candidates, 66 absolute and 34 relative. To further prioritize and select the most promising candidates, each approach's selected genes were crosschecked for their performance in the other two techniques. Candidates were considered highly promising if they had two methods supporting them. Additionally, since many of the genes could not be tested in one or more of the approaches, candidates could still be considered highly promising if supported by one method. However, they could not be tested in both methods. For example, Os02g0635600 ranked highest in Q22 (harvest index in absolute HNT) based on ortholog to known causal genes using QTG-Finder2, but this gene was not present in the full gene regulatory network and was not detected in the transcription data (Table 3). Therefore, we only penalized against genes that did not perform well where they could be tested. A total of 56 genes are identified as the “high-priority” gene candidates based on this method, 35 genes from the loci identified in the absolute HNT or control and 21 genes from the relative analysis (Supplementary Table S4). Seventeen candidate genes were identified as strong candidates in two of the three evaluations, 12 in the absolute and 5 in the relative analysis. An additional 23 candidate genes were selected as priority candidates in absolute HNT or control based on one criterion but could not be tested in either of the other two methods. Similarly, 16 additional priority candidates identified by one method, but not testable in the other two were identified from the loci associated with the relative change between control and HNT (Supplementary Table S4). A detailed list of the transcription factor (TF) for each candidate gene is given in Supplementary Table S5.

**Table 3:** List of genes closely related to absolute traits under HNT and relative across experiments.

SNP	Trait	Exp	RAP DB_ID	In_DE_Set	Rank	In_Network	Description
**Genes closely related to absolute traits under HNT**							
Chr1pos22255654.1	YPP	E1	Os01g0576100	No	1	No	Helix-loop-helix DNA-binding domain containing protein
Chr2pos31622043.1	YPP/HI	E1	Os02g0806900	Yes	2	Yes	Pyridoxal phosphate-dependent transferase, major region, subdomain 1 domain containing protein
Chr2pos31622043.1	YPP/HI	E1	Os02g0807800	No	1	No	Hypothetical conserved gene
Chr11pos23922165.1	YPP	E1	Os11g0615800	No	1	No	DNA repair protein RAD51, Homologous pairing
Chr11pos23922165.1	YPP	E1	Os11g0615900	No	2	No	Conserved hypothetical protein
Chr8pos1149355.1	YPP	E2	Os08g0120900	No	2	No	Protein of unknown function DUF247, plant family protein
Chr2pos4831761.1	HWT	E1	Os02g0186100	No	2	No	Hypothetical conserved gene
Chr4pos4550145.1	HWT	E2	Os04g0166500	No	2	No	Hypothetical conserved gene (encodes a secreted putative subtilisin-like protease)
Chr4pos4550145.1	HWT	E2	Os04g0166600	Yes	1	Yes	Similar to OSIGBa0136O08-OSIGBa0153H12.4 protein
Chr2pos25528306.1	HI	E1	Os02g0635600	No	1	No	Tyrosine protein kinase domain containing protein
Chr11pos3631830.1	HI	E1	Os11g0173550	No	2	No	Hypothetical conserved gene
Chr11pos3631830.1	HI	E1	Os11g0173750	No	1	No	Similar to Leucine Rich Repeat family protein, expressed
Chr1pos4312404.1	HI	E2	Os01g0180900	Yes	2	Yes	2-oxoglutarate and iron-dependent oxygenase, Starch accumulation
Chr1pos4312404.1	HI	E2	Os01g0182700	No	1	No	Similar to WRKY12
Chr1pos39165647.1	CHALK	E2	Os01g0899800	Yes	2	Yes	Pathogenesis-related transcriptional factor and ERF domain containing protein
Chr2pos33430148.1	CHALK	E2	Os02g0786500	–	1	–	Similar to PR-1a pathogenesis related protein (Hv-1a) precursor
Chr4pos20331507.1	CHALK	E2	Os04g0411800	No	2	No	Similar to H0717B12.5 protein
Chr4pos20331507.1	CHALK	E2	Os04g0412300	No	1	No	Glycoside hydrolase, family 17 protein
Chr3pos16794259.1	GL	E2	Os03g0409100	Yes	4	Yes	PUA-like domain domain containing protein
Chr5pos5366489.1	GW	E2	Os05g0187100	–	1	–	Cytosolic hexokinase, Sugar signaling and metabolism
**Genes closely related with relative traits values**							
Chr1pos22255654.1	YPP	E1	Os01g0577300	No	3	No	bHLH protein, Regulation of anthocyanin synthesis in the leaf sheath, (Nipponbare: nonfunctional)
Chr1pos22255654.1	YPP	E1	Os01g0576100	No	3	No	bHLH protein, Regulation of anthocyanin synthesis in the leaf sheath, (Nipponbare: nonfunctional)
Chr2pos34468097.1	YPP	E1	Os02g0807800	No	1	No	Hypothetical conserved gene
Chr3pos15905968.1	YPP	E1	Os03g0394900	No	2	No	Similar to nodulation protein-related
Chr5pos25446637.1	YPP	E2	Os05g0513200	Yes	3	Yes	NLI interacting factor domain containing protein
Chr5pos29593994.1	HWT	E2	Os05g0594200	Yes	2	Yes	Similar to Cation/proton exchanger 1a
Chr5pos29593994.1	HWT	E2	Os05g0595200	No	1	No	Allergen V5/Tpx-1 related family protein
Chr8pos25495868.1	HWT	E2	Os08g0514600	No	1	No	Hypothetical conserved gene
Chr8pos25495868.1	HWT	E2	Os08g0514100	No	2	No	Legume lectin, beta chain domain containing protein
Chr8pos4667697.1	HWT	E1	Os08g0179000	No	1	No	Protein kinase, catalytic domain domain containing protein
Chr8pos4667697.1	HWT	E1	Os08g0179150	No	1	No	Hypothetical conserved gene
Chr11pos3631830.1	HI	E1	Os11g0173750	No	1	No	Similar to Leucine Rich Repeat family protein, expressed
Chr11pos3631830.1	HI	E1	Os11g0173550	No	2	No	Hypothetical conserved gene
Chr7pos6689342.1	HI	E2	Os07g0220466	No	1	No	Similar to H0315A08.1 protein
Chr2pos18751285.1	CHALK	E2	Os02g0517564	No	1	No	Similar to Ribose-phosphate pyrophosphokinase 1
Chr3pos15967569.1	CHALK	E1	Os03g0395100	No	1	No	Protein phosphatase 2C domain containing protein
Chr8pos20389531.1	CHALK	E2	Os08g0425100	Yes	2	Yes	Dynamin family protein
Chr8pos20389531.1	CHALK	E2	Os08g0424100	No	1	No	Similar to carbonic anhydrase
Chr3pos33471970.1	GL	E2	Os03g0802100	Yes	1	Yes	Similar to F-box domain containing protein, expressed
Chr1pos3609856.1	GW	E2	Os01g0170300	Yes	1	Yes	Serine/threonine protein kinase-related domain containing protein
Chr7pos25293594.1	GW	E2	Os07g0614000	No	1	No	Six-bladed beta-propeller, TolB-like domain containing protein

Genes located within the vicinity of significant GWAS sites were used to identify closely connected genes using the Network analysis. A list of annotated genes around the cMTAs is given in the Supplementary Table S2. E1-walk-in chambers (Experiment 1) and E-2—field-based heat tents (Experiment 2). The In_DE_Set, Rank, and In_Network columns correspond to the different gene candidate prioritization methods. These three columns indicate the gene’s presence in the DE the gene’s rank in its respective loci and network datasets, respectively.

*YPP* grain yield, *HWT* 100-seed weight, *HI* Harvest index, *CHALK* chalkiness, *GL* grain length, *GW* grain width.

## Discussion

Increasing tolerance to HNT while simultaneously minimizing yield and quality losses is an emerging goal in rice breeding. A night temperature of > 27 ℃ has been used to determine differential physiological, biochemical, and agronomic responses both under controlled^19,53^ and field experiments^11,13,32^, using a limited number of genotypes [two^11^ to 36^33^]. Here, we carried out stage-targeted phenotyping of *indica* diversity panel both under controlled-environmental and field conditions (Supplementary Fig. S1), which allowed us to identify genetic loci associated with key phenotypic traits that determine HNT responses (Table 2).

### Phenotypic variation explains underlying trait correlations and physiological responses under HNT

Our study involving rice diversity panel (entries from different rice-growing regions) recorded a significant decline in yield, 100-seed weight, and increased chalkiness under prolonged HNT stress (Fig. 2), in both controlled environment and field studies, supporting previous findings^5,11,13^. A more significant reduction in grain yield and 100-seed weight, at least in part, is known to be related to reduced carbohydrate availability due to higher night-time respiration or reduced assimilates supply to sink or a combination of both, under HNT in rice^13^. HNT stress during grain development terminates or reduces the activity of a series of enzymes involved in starch synthesis in the endosperm, negatively affecting grain size under HNT in rice^13,35^ and other crops^14^. For example, a significant correlation between changes in the fructose-6-phosphate content of the panicles and the yield reduction in rice emphasized the significant metabolic changes in the sink during grain development in response to HNT^35^. Thus, minimizing yield losses and quality deterioration caused by HNT during grain filling is becoming extremely important to sustain global rice production and end use quality.

Unlike HDT stress at anthesis, post-flowering HNT reduces grain yield and quality by lowering grain weight caused by heat-induced premature senescence^14^ and quicker break‐down of stored assimilates during the night^13,32,35^. In particular, heat stress-accelerated chlorophyll degradation, inhibition of chlorophyll biosynthesis^54^, and loss of carbon already fixed by daily photosynthesis^55^ are key aspects that limit the source capacity during grain filling. The depletion of carbohydrates or imbalance with the source (decrease in photosynthesis and increase in respiration) during grain filling exhausts the assimilate reserves that are particularly needed for grain development under heat stress^14,34,56^. Similar to the impacts of HNT stress, low solar radiation also interferes with starch accumulation during grain filling, which significantly affects the seed weight and market value (quality) of rice^57^. An increase in average night-time VPD seems to be coupled with warmer night and lower relative humidity (Fig. 1b). Such an increase in night VPD has been shown to impact the plant productivity^58^. Hence, further studies are needed to disentangle the HNT vs. low solar radiation, differential VPD levels under HNT scenarios and their combined effect on yield and grain quality losses in rice.

Interestingly, canopy functional stay-greenness is known to have the potential to enhance radiation use efficiency and photo-assimilates supply by improving the longevity of photosynthetic machinery during post-anthesis. We hypothesize that the canopy's extended ability to generate additional assimilates under HNT could potentially fill the carbon gap created by increased carbon losses due to higher night respiration in response to HNT. Therefore, exploring the relevance of functional stay green in combination with an efficient transfer of photo-assimilates (water-soluble carbohydrates) from source to sink is proposed to minimize the HNT-induced yield and quality losses during grain filling in rice.

### Comparison of GWAS revealed common loci for phenotypic traits across treatments

Grain size/shape and chalkiness are two crucial components that determine rice grain quality^59^, with increased chalkiness having a significant negative impact on the economic returns^20^. Both grain size and chalkiness are polygenic quantitative traits^60^. The high genetic and phenotypic correlations among measured traits across treatments, particularly for seed weight and grain size, indicate strong potential surrogate traits for breeding HNT tolerance in rice. A weak correlation was observed between controlled environment and field for some traits, which might be due to one or more factors (thermal amplitude [Fig. 1b], low light condition, relative humidity) differing between these environments^23,30^. In spite of these differences, several GWAS loci were in the genic region or close to previously reported genes controlling grain shape traits, e.g., *GS3*^51,52^ and *GW5/qGSW5*^49,50,61^ across treatments. This indicates that (1) GWAS is an effective way to identify putative genes/target regions for HNT tolerance in rice and (2) exhibited the presence of strong and common genetic regulation across control and HNT in rice (Fig. 4). For example, a strong association for grain width was identified on chromosome 5 under both control and HNT conditions, which is in the region of a well-characterized gene, *GW5* (Os05g0187500, encoding a calmodulin-binding motif family protein) that regulates grain width in rice^50^ (Fig. 4c,d). Another prominent peak on chromosome 3 (Chr3pos16794259.1) associated with the gene, *GS3* (Os03g0407400, a regulator of grain size and organ size), was detected for grain length across treatments (Fig. 4f,g). Co-localization of GWAS loci for grain size (length or width) variations under control and warmer nights suggest that tolerance to HNT stress for grain size is likely to have common regulators in rice (Fig. 4). Another cMTA was identified on chromosome 3 (Q28) for grain chalkiness, which is also near the *GS3,* indicating that the molecular mechanisms of grain chalkiness in rice might be rather complex than the grain size^59^. Therefore, further functional studies are needed to explore the association of grain size and chalkiness genes in regulating rice grain weight and quality under heat stress conditions.

For instance, one genomic region on chromosome 2 (31.4–31.9 Mb) associated with Q2, Q3, Q8, and Q12 was shown to control yield and 100-seed weight either under control or HNT (Supplementary Fig. S2). Loci (Q3 and Q8) identified under control and HNT grain yield were close (25 kb) to the trehalose-6-phosphate phosphatase gene (TPP7, Os02g0753000), which is shown to display significant improvement in abiotic stress tolerance in plants^62^. In Maize, the overexpression of trehalose-6-phosphate phosphatase in-ears increased yield by increasing both kernel set and harvest index in drought stress conditions^63^. On the other hand, increased trehalose concentration was recorded after four hours of heat stress (at 40 °C) in *Arabidopsis thaliana*^64^. The locus (Q8) associated with yield under HNT was close (32.2 kb, Os02g0753800) to a calcium-binding protein (Supplementary Table S3), which is found to enhance heat stress tolerance by modulating the reactive oxygen species (ROS) production, particularly H_2_O_2_ and reproductive organ development under different abiotic stresses^65,66^. A similar family gene (OsANN10) has been mapped for the heat stress tolerance index in rice^67^. A defined role for these genes in combating heat stress at reproductive and grain-filling has not been fully explored.

Despite the variable number of genotypes and different experimental conditions, our phenotypic correlations and co-localized GWAS loci exhibited the presence of strong and common genetic regulation across treatments in rice. A genomic region associated with the relative grain length on chromosome 3 (33.47) co-localized with locus influencing absolute spikelet fertility under high temperature^68^ and relative spikelet fertility^69^. Two cMTAs for absolute grain yield under HNT on chromosome 1 (Q5, Chr1pos22255654.1) and chromosome 2 (Q6, Chr2pos34468097.1) were related to genomic regions for relative grain yield in experiment 1 (Table 2). Similarly, a common MTA was observed between harvest index under HNT (Q25, Chr11pos3631830.1) and relative harvest index in experiment 1 (Table 2). Therefore, further characterization of loci (31.46 to, 34.46 Mb on chromosome 2 and 33.47 on chromosome 3) would be interesting to decipher the role of these pleiotropy regions for enhancing heat stress resilience (both day-time and night-time) in rice. Relatively little is known about the interaction between high day and night temperature stress on grain yield and quality^70^. Identifying genomic regions associated with combined stress tolerance would set a stage to breed rice with improved heat stress tolerance.

A limited number of shared loci between relative and absolute phenotype were observed across treatments suggesting that mapping genetic loci based on stress index (stress effects relative to control) may not always reflect the true stress tolerance^68^. While comparing the relative change in phenotypes between control and HNT allows the evaluation of sensitivity to HNT stress across all phenotypic ranges. In addition, evaluating the candidate genes in these loci may provide firsthand targets for pathways that alter adaptation to HNT stress. Nevertheless, quantifying stress tolerance by combining both absolute and relative traits would help develop rice accessions with greater productivity and adaptability under stress. The identified loci can be used as a starting point for future candidate gene validation and breeder-friendly markers development to enhance HNT tolerance in rice.

### Refinement of candidate genes by available molecular data

Although the possible genetic determinants of phenotypic trait variations in response to stress have been effectively determined based on GWAS in crops, detecting their functional relevance or prioritizing candidate genes is a severe bottleneck^46^. Using available prior data, we refined the possible candidate genes associated with the traits for each genomic region through three independent methods: evaluating the transcriptional response of the genes in each locus to HNT, determining the connectedness of each gene to transcriptional regulators in a gene regulatory network, and determining the orthology to genes previously identified as causative in other studies. Validation of the causal gene for each GWAS-identified locus is a key component to move forward. However, it is not practical to experimentally test all the genes that overlay the genomic regions. Therefore, there is a need to prioritize the target genes. Previous genetic mapping or GWAS studies have routinely used currently available literature to decide on the potential candidates to validate based on annotations and known functions. This approach, although widely used, introduces bias at different levels^45,46^. Hence, developing new approaches that reduce this bias would be beneficial. The three methods used to find top candidates from this study demonstrate integrating multiple ways to prioritize candidates for further validation. One limitation of this approach is that not all candidate genes could be tested for many of these datasets. For example, genes not on the Agilent rice 4 × 44 K microarray were not included in the gene regulatory network. Likewise, genes not expressed in the tissue evaluated for HNT response were not included in the transcriptional analysis. However, as data resources extend and more data becomes available in accessible formats, these approaches will become even more reliable for narrowing down the candidate genes to test for causality.

## Conclusion

The current study is the first report investigating the natural variation of rice genotypes to HNT using walk-in chambers and field-based heat tents. The observed significant positive correlations (Fig. 3) and common GWAS loci across treatments (Fig. 4) for grain size indicate that selection for higher phenotypic values from the control conditions could be a proxy for high yields under HNT. Advanced gene sorting methods combined with GWAS allowed us to narrow down the candidate region to potential genes controlling yield and related traits across control and HNT. Exploring the genetic regulation of functional stay green and source-sink carbohydrate relationships under night temperature is an exciting area for further research. Our findings provide insights towards integrating multi-disciplinary approaches such as physiological, genomic, and network algorithms to identify possible causal loci/genes. The identified favorable alleles for HNT tolerance would help develop molecular markers to support ongoing heat stress breeding programs in rice.

## Supplementary Information

Supplementary Table S1.

Supplementary Table S2.

Supplementary Table S3.

Supplementary Table S4.

Supplementary Table S5.

Supplementary Table S6.
