# Supplementary material for: Genome-wide association study and gene network analyses reveal potential candidate genes for high night temperature tolerance in rice
Source: Sci Rep. 2021 Mar 24;11:6747. doi: 10.1038/s41598-021-85921-z (PMC7991035; doi:10.1038/s41598-021-85921-z)
Supplement: Supplementary file 6 — Supplementary Table S6. [file 41598_2021_85921_MOESM6_ESM.docx]

**Title – Genome-wide association study and gene network analyses reveal potential candidate genes for high night temperature tolerance in rice**

**Authors** - Raju Bheemanahalli^1,2,#^, Montana Knight^3^, Cherryl Quinones^1,4^, Colleen J. Doherty^3^, S.V. Jagadish Krishna^1,2^*

**Affiliations**

^1^International Rice Research Institute, DAPO Box 7777, Metro Manila, Philippines

^2^Department of Agronomy, Kansas State University, Manhattan, KS 66506, USA

^3^Department of Molecular and Structural Biochemistry, North Carolina State University, Raleigh, NC 27695, USA

^4^Arkansas Biosciences Institute, Arkansas State University, State University, P. O. Box 419, Jonesboro, AR, 72467 USA

^#^Present address: Department of Plant and Soil Sciences, Box 9555, 117 Dorman Hall, Mississippi State University, Mississippi State, MS 39762, USA

**Email address:**

Raju Bheemanahalli: [rajubr@pss.msstate.edu](mailto:rajubr@pss.msstate.edu) (ORCID 0000-0002-9325-4901)

Montana Knight: [mknight@ncsu.edu](mailto:mknight@ncsu.edu)

Cherryl Quinones: [cherryl.quinones@smail.astate.edu](mailto:cherryl.quinones@smail.astate.edu)

Colleen J. Doherty: [cjdohert@ncsu.edu](mailto:cjdohert@ncsu.edu) (0000-0003-1126-5592)

S.V. Jagadish Krishna: [kjagadish@ksu.edu](mailto:kjagadish@ksu.edu) (0000-0002-1501-0960)

***Corresponding author**

S.V. Krishna Jagadish

Department of Agronomy

2004 Throckmorton Plant Sciences Center,

1712 Claflin Road, Manhattan, Kansas 66506-5501

E-mail - [kjagadish@ksu.edu](mailto:kjagadish@ksu.edu)

**Supplementary Figure S1.** Schematic representation of materials and methods followed to explore HNT stress tolerance in rice using controlled walk-in chambers (Experiment 1) and field-based heat tents (Experiment 2) facilities. In experiment 1 (walk-in chambers), temperature treatments included control night temperature (CNT) and high night temperature (HNT), starting from panicle initiation to physiological maturity. In experiment 2, genotypes were exposed to two different night temperature treatments i.e., control night (CNT) and high night temperature (HNT) with common day-time ambient temperature across treatments.

**Supplementary Figure S2.** Manhattan plots for grain yield (A-F), 100-seed weight (G-L), and harvest index (M-R) in Exp. 1 and Exp. 2. Detected significant MTAs in the study are given in the Supplementary Table S1. In experiment 1 (walk-in chambers), temperature treatments included control night temperature (CNT) and high night temperature (HNT) and in experiment 2, two-night temperature treatments i.e., control night (CNT) and high night temperature (HNT) were imposed with common day-time ambient temperature across treatments. The list of candidate marker-trait associations (cMTAs) considered in the study are plotted or presented in Table 2. Annotated genes around the cMTAs are given in Supplementary Table S2. Relative = high night temperature/control night temperature.


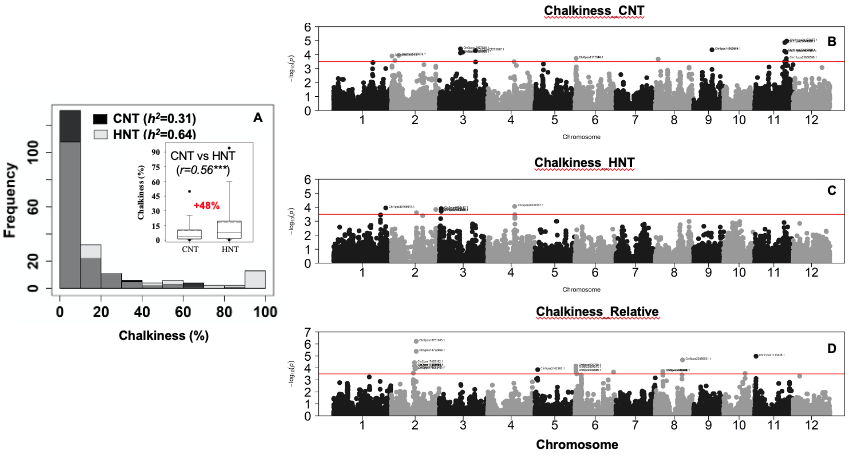


**Supplementary Figure S3.** Rice chalkiness (%) in response to control night (CNT) and high night temperatures (HNT) under common day-time temperature in Experiment 2. Manhattan plots for chalkiness (B- control night, C-high night-time temperature and D-relative values) under field-based heat tent study (Exp. 2). The marker-based heritability (*h^2^*) was obtained in the Genomic Association and Prediction Integrated Tool. The list of candidate marker-trait associations (cMTAs) considered in the study are presented in Table 2. Annotated or known genes around the cMTAs are given in Supplementary Table S2. Relative = high night temperature/ control.
